# Reviewing the Genetic and Molecular Foundations of Congenital Spinal Deformities: Implications for Classification and Diagnosis

**DOI:** 10.3390/jcm14041113

**Published:** 2025-02-09

**Authors:** Diana Samarkhanova, Maxat Zhabagin, Nurbek Nadirov

**Affiliations:** 1 National Center for Biotechnology, Astana 010000, Kazakhstan; diana.samarkhan@gmail.com; 2 Department of Orthopedics, Mother and Child Health Center, University Medical Center, Astana 010000, Kazakhstan

**Keywords:** congenital spinal deformities (CSDs), congenital scoliosis, genetic mutations, embryonic development, signaling pathways, sclerotome differentiation, somitogenesis, molecular classification, bone morphogenetic proteins (BMPs)

## Abstract

Congenital spinal deformities (CSDs) are rare but severe conditions caused by abnormalities in vertebral development during embryogenesis. These deformities, including scoliosis, kyphosis, and lordosis, significantly impair patients’ quality of life and present challenges in diagnosis and treatment. This review integrates genetic, molecular, and developmental insights to provide a comprehensive framework for classifying and understanding CSDs. Traditional classification systems based on morphological criteria, such as failures in vertebral formation, segmentation, or mixed defects, are evaluated alongside newer molecular-genetic approaches. Advances in genetic technologies, including whole-exome sequencing, have identified critical genes and pathways involved in somitogenesis and sclerotome differentiation, such as *TBX6*, *DLL3*, and *PAX1*, as well as key signaling pathways like Wnt, Notch, Hedgehog, BMP, and TGF-β. These pathways regulate vertebral development, and their disruption leads to skeletal abnormalities. The review highlights the potential of molecular classifications based on genetic mutations and developmental stage-specific defects to enhance diagnostic precision and therapeutic strategies. Early diagnosis using non-invasive prenatal testing (NIPT) and emerging tools like CRISPR-Cas9 gene editing offer promising but ethically complex avenues for intervention. Limitations in current classifications and the need for further research into epigenetic and environmental factors are discussed. This study underscores the importance of integrating molecular genetics into clinical practice to improve outcomes for patients with CSDs.

## 1. Introduction

Congenital spinal deformities (CSDs) are disorders of the musculoskeletal system, characterized by abnormal development of the vertebral column at the embryonic stage. Morphologically, CSDs are divided into scoliosis, kyphosis, and lordosis. Congenital scoliosis (CS) is a lateral displacement of the spine that forms during the period of intrauterine development. A meta-analysis covering 101,548 children and adolescents shows that the prevalence rate of congenital scoliosis is about 0.215% [1]. There is a lack of information on the incidence rate since CS is a rare spinal deformity. Worldwide, the incidence rate of CS is between 0.5/1000 and 1/1000 births [2]. In a recent study conducted using the Korean Statistical Information Service database, the incidence of CS was 3.08 per 100,000 over the 5-year period [3,4]. The other two types of CSD based on morphology are kyphosis, backward curvature of the thoracic region, and lordosis, forward curvature of the lumbar and cervical spine, which are less common than congenital scoliosis [5].

Although congenital spinal anomalies are rare, they cause severe progressive pain syndromes, complicating the lives of patients with CSDs. Therefore, it is necessary to analyze the causes of congenital spinal deformities, which could allow early diagnosis and management to improve the lives of people with such conditions. Embryonic spine development is a complexly organized series of events. Gaining knowledge of these features will help to understand the pathogenesis of congenital spinal anomalies. Congenital spinal deformities represent a unique challenge in medical research and clinical practice, as they encompass a broad spectrum of genetic, molecular, and environmental factors. This review aims to integrate recent findings on the genetic and molecular underpinnings of these deformities, offering a comprehensive framework for their classification, diagnosis, and potential therapeutic strategies. The ultimate goal is to provide clinicians and researchers with an enhanced genetic understanding of the pathogenesis of CSDs and to equip them with the tools necessary to optimize patient outcomes.

## 2. Classification of Congenital Spinal Deformities

### Nature of Vertebral Development Failure

Proper diagnosis, treatment, and research of congenital anomalies of the spine require the classification of CSDs. These deformities can be classified differently based on various criteria. CSDs can be classified based on the nature of vertebral development failure in the following three groups: formation failure, segmentation failure, and mixed type [6]. Formation failure includes wedge-shaped vertebrae (WSV), hemivertebrae (HV), butterfly vertebrae (BV), and vertebral aplasia (VA), characterized by the partial or complete absence of vertebral elements. WSV is characterized by asymmetrical development, in which one side of the vertebra is smaller than the other. The failure of one-half of the vertebral body to develop results in the formation of HV. The total absence of the vertebral body is known as the VA.

**Figure 1:**
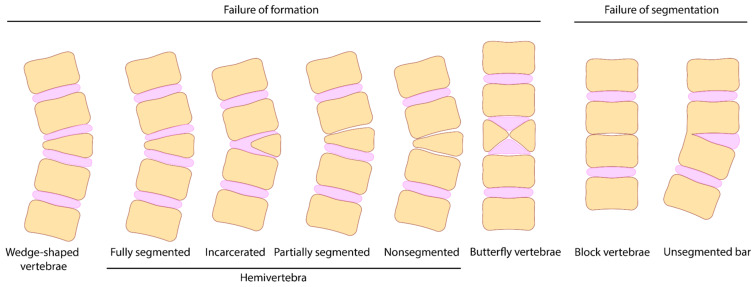
Classification of the spinal deformities based on the nature of vertebral development failure.

Segmentation failure is an absence of the segmented structure of the vertebral column due to partial or complete fusion of two or more vertebrae. This failure developed at the embryonic stage of the spinal column segmentation, which includes unilateral unsegmented bars and block vertebrae (Figure 1). Fusion of two or more adjacent vertebrae in one side identifies a unilateral nonsegmented spine, which results in vertebra rigidity and congenital scoliosis showing challenges for surgical treatment [7]. Block vertebra is the result of a segmentation failure associated with the fusion of two or more vertebrae due to a fibrous intervertebral junction or complete absence of the discoid structure [8].

The third classification group of CSDs is the mixed type, which includes cases with formation and segmentation failures simultaneously or anomalies that do not fit their descriptions. The vertebral formation anomalies are extremely variable in terms of severity, extent, and rate of progression of the deformation. The classification of CSDs into formation failure, segmentation failure, and mixed type was described in the late 20th century and was accepted worldwide [9]. However, the lack of resources and technologies used for the study and description of CSDs indicates the need for a new classification.

Later, in 2009, Kawakami and colleagues published a new classification of CSDs derived from the analysis of three-dimensional (3D) computer tomography images [9]. Novel classification considered 4 types of CSDs, including solitary simple (SS), multiple simple (MS), multiple complex (MC), and segmentation failure (SFK) (Table 1). SS describes an abnormal vertebra with a formation disorder, including HV, WSV, BV, and others. MS involves multiple anomalies of vertebrae resulting in CSD occurrence and is not related to the anterior and posterior structure relationship. A combination of HV, WSV, BV, discrete, adjacent, and other types constitutes the MS type. MC covers formation defects with some segmentation defects, while SFK only covers segmentation failure.

In 2018, Kawakami et al. proposed a modified classification for CSDs that categorizes these defects into formation failure, coupling failure, segmentation failure, and mixed failure [10]. Formation failure and segmentation failure are consistent with the same characteristics described by Winter et al. [6]. Novel classification differs from previous systems by the introduction of a coupling failure type. Coupling failure is characterized by the false coupling of somites resulting in the formation of contralateral hemivertebrae. Previously, such defects were classified as formation failure. Mixed failure covers coupling failure with segmentation or formation defects.

It is important to note that CSDs are highly variable, and their classification based on the nature of vertebral development failure may not be complete and precise. Limitations of the classifications of Winter et al. [6] and Kawakami et al. [9,10] are the presence of a group, such as ‘mixed type’ and ‘mixed complex’, which include cases that could not be classified into other categories due to a combination of defects. The presence of ‘mixed’ type categories poses challenges in the planning of treatment, prognosis, and research. Therefore, it is necessary to develop a classification system that reflects the complexity of CSDs.

## 3. Embryonic Development of the Vertebral Column

Congenital spinal deformities are serious conditions that require a comprehensive approach to diagnosis and treatment. Congenital scoliosis and other CSDs are formed during embryonic development. Understanding the processes of embryonic development of the spine and the risk factors that contribute to the occurrence of these pathologies is key to timely prevention and effective treatment. There are three main stages of the development of vertebrae: mesenchymal, cartilaginous, and bony stages. Vertebrae development involves notochord development, somite differentiation, sclerotome migration, chondrification, and ossification of the vertebrae [11]. The vertebrae development starts with notochord formation, where the paraxial mesoderm differentiates into the axial skeleton [12]. Somites, paired segmented structures that give rise to vertebrae, are formed from the paraxial mesoderm. The ventral region of the somite forms the sclerotome, which differentiates into vertebrae and ribs, and the dorsal region gives rise to the dermomyotome that forms the dermatome and myotome, forming the skin and muscles [13].

**Figure 2:**
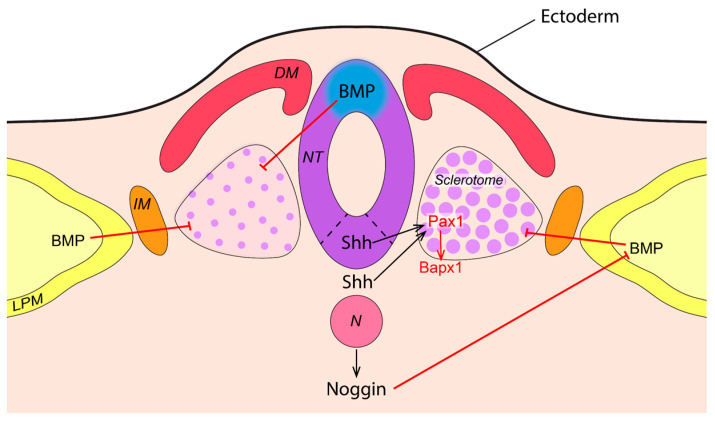
SHH and BMP signaling in sclerotome development. SHH protein initiates sclerotome formation and its transformation into cartilage after secretion from the notochord and neural tube floor plate. During early sclerotome development, SHH is antagonized by BMP signals from the LPM. Sclerotome development is supported by the secretion of Noggin from the notochord, which inhibits BMP and promotes cartilage development in conjunction with SHH. PAX1 and PAX9 activate the *BAPX1* gene, which is necessary for axial skeleton development. DM—dermomyotome, IM—intermediate mesoderm, N—notochord, NT—neural tube, LPM—lateral plate mesoderm.

Sclerotome formation is regulated by the Sonic hedgehog (SHH) protein released from the neural tube floor plate and notochord [14,15]. Sclerotome cells migrate around the notochord and neural tube during the fourth week of embryonic development, which subsequently leads to the formation of cartilaginous tissue. SHH is expressed in the vertebral part of the developing vertebrae and triggers the PAX1 transcription factor (TF), which is crucial for sclerotome cells to acquire ventral and dorsal identities [16,17]. The synergistic work of PAX1 protein with other TFs, such as PAX9 and MFH1 proteins, leads to axial skeleton development (Figure 2). PAX1 protein is responsible for the differentiation of sclerotome cells into chondrocytes that form cartilage and somite segmentation [18]. On the dorsal side of the forming vertebrae, bone morphogenic proteins (BMPs) are involved in the bone and cartilage formation [19]. The result of *SHH* and *BMP* signaling is the formation of the dorsal and ventral sides of the vertebrae.

The second stage of vertebral development is the cartilaginous stage, in which chondrification centers appear in the 6th week of embryonic development. The final stage of spinal development is the skeletal stage, which begins at 8 weeks and is not completed until the mid-twenties. Between the 5th and 8th weeks of gestation, sclerotome cells differentiate into chondroblasts. This process of formation of cartilaginous models of the vertebrae is tightly controlled by *HOX* genes. *HOX* gene regulation is responsible for anterior–posterior positional information during vertebrate development and correct regionalization of the vertebrae in the vertebral column [20,21]. *HOX* genes provide spatial identity to developing tissues throughout the embryo. Mutations in these genes alter tissue identity within their expression domains, often in the anterior region.

To sum up, *HOX* genes provide regional identity to developing vertebrae, while SHH signaling controls PAX1 protein expression in the sclerotome. CSDs can result from mutations in any of these genes, which can cause a chain reaction that disrupts the balance of these interactions. Altered *PAX1* expression levels caused by disrupted SHH signaling may result in defects in sclerotome differentiation. Similarly, changes in *HOX* gene expression may affect vertebral size, shape, and alignment by influencing the interpretation of PAX1 and SHH signals during vertebral development. The complex interactions between these genes highlight the importance of carefully regulating them during embryogenesis to avoid congenital defects.

## 4. Genetic Basis of Spinal Deformities

Congenital spinal deformities are usually the result of genetic mutations, such as those seen in Marfan syndrome [22], Klippel–Feil syndrome (KFS) [23], spondylocostal dysostosis [24], VACTERL/VATER association [25], Alagille syndrome [26], Ehler–Danlos syndrome [27], and others. The mutations responsible for these disorders disturb the individual gene or gene cascades involved in the development and proper functioning of organs and tissue unrelated to the musculoskeletal system. Therefore, it is necessary to classify CSDs based on mutations in genes that are associated with these disorders.

Advances in molecular genetic analysis, including whole-exome sequencing, have enhanced the ability to identify the genetic basis of congenital disorders. Gene mutations that affect the entire process of embryonic development can result in CSDs. Numerous genes and signaling pathways, such as fibroblast growth factor (FGF), wingless-related integration site (Wnt), Notch, and others, are involved in various stages of vertebrae development [28]. These genes frequently switch on and off and regulate the mechanisms of organ and tissue formation and development. These genes are in turn regulated by a number of regulatory genes. These gene cascades can be subject to breakdowns due to gene mutations leading to disruptions in developmental processes.

Classification based on the nature of vertebral development failure does not fully reflect the complexity of CSDs. A new classification of spinal deformities can be suggested based on particular genetic abnormalities that interfere with critical pathways involved in spinal development discovered by whole-exome sequencing. Therefore, two categories of classification can be outlined for CSDs. One category classifies based on the stage of vertebrae development, developmental stage-specific malformation of the spine; another one relies on signaling pathways involved in vertebral development, signaling pathways in CSDs.

## 5. Classification of CSDs Based on Developmental Stage Specificity

There are numerous key genes involved in vertebrae development that have been identified thanks to recent advances in whole-exome sequencing [29]. Developmental defects are usually caused by alterations in the genetic components of key developmental stages such as somitogenesis and sclerotome differentiation. A thorough understanding of the genetic basis of these problems will make it possible to selectively diagnose and treat developmental phases affected by stage-specific mutations. CSDs can be categorized according to the developmental stages that are interrupted by specific mutations and result in spinal abnormalities. Two main categories of this developmental, stage-specific classification are somitogenesis-related and sclerotome differentiation disorders.

### 5.1. Somitogenesis-Related CSDs

The first category of CSD classification is somitogenesis-related disorders. Somitogenesis is a process of the formation of somites. This process is regulated by FGF, Notch, and Wnt signaling within presomitic mesoderm cells [30]. Somites, segmented blocks of mesoderm in the embryo, are formed from the paraxial mesoderm and undergo resegmentation to give rise to vertebrae, ribs, and associated musculature [31]. This segmentation process is disturbed by mutations affecting genes involved in somitogenesis, which leads to different CSDs, such as spondylocostal dysostosis (SCD) and CS. There are different genes involved in somitogenesis.

During somitogenesis, TBX6 protein controls the differentiation and segmentation of paraxial mesoderm [32]. About 10% of patients with congenital scoliosis had a heterozygous nonsense/frameshift mutation in the *TBX6* gene or a heterozygous deletion on chromosome 16p11.2. It should be noted that patients with *TBX6* heterozygous null mutation had the same haplotype (T-C-A) on the other allele, identified by the following SNPs: rs2289292, rs3809624, and rs3809627 [33,34]. Mutations in *TBX6*, which is crucial for somite development, cause obvious skeletal abnormalities. Another gene associated with somitogenesis is *DLL3*, which encodes a ligand for the Notch signaling pathway. In research analyzing the genetic basis of SCD, there were observed nonsense, missense, frameshift, splicing, and in-frame insertion mutations in exons 4–8 of the *DLL3* gene [35]. In a recent study, a novel homozygous nonsense variant c.535G>T in the *DLL3* gene was observed in a Pakistani family with SCD [36]. The *MEOX1* gene is expressed in the presomitic mesoderm, and mice with a mutation in this gene show vertebrae and rib bone defects derived from the sclerotome, a ventral part of a somite [37]. In a patient with KFS, a homozygous transition c.664C>T was observed in the *MEOX1* gene, which leads to the formation of a premature stop codon [38].

### 5.2. Sclerotome Differentiation CSDs

The second group of developmental stage-specific CSD classifications is the sclerotome differentiation disorders. After somitogenesis, somites are subjected to differentiation to form a sclerotome, which subsequently leads to the epithelial–mesenchymal transformation of somite cells and their detachment from the epithelial somite [39]. There are different genes associated with early sclerotome development, such as *PAX1*, *PAX9*, and *BAPX1*. *PAX1* and *PAX9* genes encode transcription factors that control the differentiation of vertebrae from sclerotome [17]. It was found that *Pax1/Pax9* double mutant mice lack vertebral column bones and proximal ribs, intervertebral disks, and the sclerotomal cells that contribute to these structures are unable to undergo chondrogenesis, a cartilage formation process [40]. In turn, PAX1 and PAX9 proteins are required for the expression of BAPX1 involved in the initiation of chondrogenic differentiation [41]. Overexpression of PAX1 protein can substitute SHH, which activates BAPX1, to initiate chondrogenic differentiation. The role of the *MEOX1* gene is not limited to the somitogenesis stage only; it was observed that this gene is required for proper sclerotome polarity [42]. The *MEOX1* gene has a dual role in somitogenesis and sclerotome differentiation, indicating its importance in vertebrae development.

Classification of CSDs into somitogenesis-related disorders and sclerotome differentiation disorders offers a genetically based approach. This approach helps to make a more precise classification, which can offer a new therapeutic approach to the treatment of central nervous system diseases. Physicians can anticipate spinal deformities at an early stage, for example, by testing on *TBX6*, *DLL3*, and *MEOX1* gene mutations, which provide personalized treatment. Abnormalities in sclerotome differentiation can be detected by identifying mutations in *PAX1*, *PAX9*, and other genes associated with this stage of vertebrae development. Identifying specific gene mutations allows for accurate diagnosis, which in turn will enable the development of targeted therapies for skeletal deformities based on the stage of development. Genes associated with CSDs have been summarized and described (Table 2).

## 6. Classification of CSDs Based on Signaling Pathways

Mutations occurring in genes not only disrupt protein translation but also affect downstream signaling pathways that are crucial for spine formation. By analyzing genes associated with CSDs and grouping them by signaling pathways, it can uncover the underlying mechanisms of CSDs and create a basis for future research in diagnostics and targeted therapy strategies. There are main signaling pathways such as Wnt/β-catenin, Notch, Hedgehog, BMP, and TGF-β involved in the vertebrae development process (Table 3) [43,44,45,46].

### 6.1. Wnt Signaling Pathway

**Figure 3:**
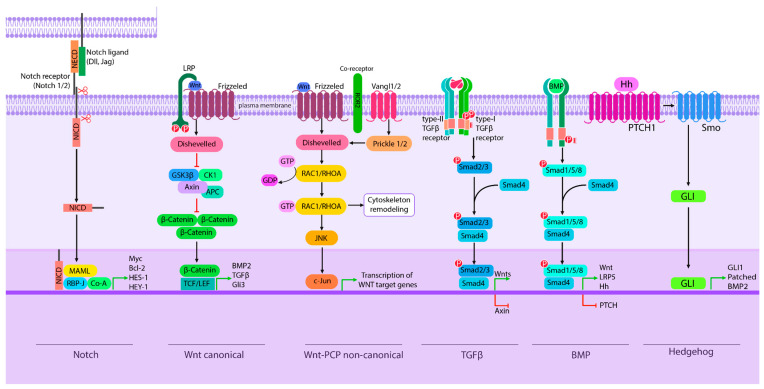
Crosstalk between Wnt, Notch, TGFβ, BMP, and Hedgehog signaling pathways. β-catenin activates the Notch signaling pathway. GSK3β phosphorylates NICD and activates Notch signaling, while Notch degrades β-catenin and negatively regulates its stability. As for the Hedgehog pathway, Wnt inhibits it by Gli3. The expression of ligands and components, such as Wnts, LRP5, Axin, BMP2, and TGF-β, is determined by TGF-β/BMP signaling and Wnt signaling, and these two signaling pathways are connected by the interaction between Smad7 and Axin.

There are two types of Wnt signaling: the β-catenin-dependent canonical pathway responsible for cell proliferation and the β-catenin-independent non-canonical pathway for cell polarity [43]. The Wnt signal stabilizes cytosolic β-catenin, which enters the nucleus and activates transcription of target genes (Figure 3). Genes such as *BAZ1B*, *KMT2D*, and *SOX9* mutations, which are associated with spinal deformities, participate in the Wnt/β-catenin pathway [47,48]. The *BAZ1B* gene was discovered to be associated with congenital vertebral fusion and associated with KFS [47]. Another gene associated with KFS is *KMT2D*, which has been identified in patients with multiple contiguous cervical fusion levels (c.13364G>A, c.3074C>T, c.8939C>T) [47]. The KMT2D protein transcriptionally regulates the beta-globin and estrogen receptor genes. Wnt signaling can inhibit SOX9 activity, which is a transcriptional factor involved in chondrogenesis, therefore it was proposed that mutation in this gene can cause congenital spinal deformities [48].

Genes correlated with non-canonical Wnt signaling are *FZD6* and *VANGL1*, and mutations in these genes were observed in CSDs [47,49]. The *FZD6* gene encodes a protein, which is a Frizzled family receptor involved in Wnt signaling [50]. Disrupted FZD6/Wnt signaling leads to a defective neural tube, which can lead to spina bifida and other congenital spinal abnormalities [49]. VANGL1 is a protein that regulates planar cell polarity [51]. Mutations in the *VANGL1* gene are found to be related to neural tube defects and spina bifida [52]. Canonical and non-canonical Wnt pathways play a critical role in the regulation of bone mass and skeletal homeostasis. Disturbed Wnt signaling leads to compromised development of neural tube and limb patterns. Moreover, the Wnt signaling pathway interacts with other signaling pathways, such as Notch, TGF-β, and BMP, which highlights its complexity in regulating various cellular processes.

### 6.2. Notch Signaling Pathway

The Notch signaling pathway, which is important in embryogenesis, controls intercellular interactions and determines the direction of cell development and differentiation [44]. This pathway is regulated by genes such as *RIPPLY2*, *TBX6*, and *JAG1*; mutations in these genes have been linked to KFS, congenital scoliosis, and Alagille syndrome [53,54,55]. Notch signaling activates the MESP2 protein, a transcriptional factor responsible for rostro-caudal patterning within a somite, which induces RIPPLY2 protein [56]. RIPPLY2 protein, a TBX6 inhibitor, acts as a transcriptional co-repressor involved in the somitogenesis and regulation of presomitic mesoderm segmentation by interacting with TBX6 protein [57]. A frameshift mutation in *RIPPLY2* was detected by exome sequencing in patients with segmentation defects of the vertebrae [53]. RIPPLY2 suppresses TBX6 expression at specific locations, which allows it to form somite boundaries accurately [58]. *TBX6* gene missense changes were observed in patients with congenital scoliosis and spondylocostal dysostosis [54]. JAG1 protein, a ligand of the NOTCH receptor, is associated with defects of vertebrae segmentation in Alagille syndrome [55]. Frameshift mutations in the *JAG1* gene cause altered protein synthesis, while deletion of this gene leads to haploinsufficiency, causing Alagille syndrome [55]. Thus, the Notch signaling pathway, associated with *RIPPLY2*, *TBX6*, and *JAG1* genes, is involved in vertebral formation, and mutations lead to segmentation defects and other skeletal dysplasias.

### 6.3. Hedgehog Signaling Pathway

Hedgehog (HH) signaling plays a crucial role in many developmental processes. Indian hedgehog (IHH), a type of HH, is expressed during endochondral ossification [45]. Furthermore, HH signaling is involved in the maintenance and repair of skeletal tissues during the postnatal period [59]. Genes such as *PAX1* and *SUFU* are involved in HH signaling, which is critical for growth plate development and chondrocyte differentiation [47,60]. PAX1 protein is a transcription factor that responds to SHH protein signaling, which is responsible for sclerotome segmentation [17]. *PAX1* gene mutations linked to CSD were found at exon 4 [60]. Another key regulator of the HH pathway is SUFU, which controls the activity of GLI transcription factors that transmit HH signals to control gene expression [61]. SUFU, in the absence of HH signals, binds to GLI proteins and keeps them in an inactive form. Even though SUFU protein is a major negative regulator of the HH pathway, a strong correlation of this gene with congenital spinal deformities has not yet been found. Some *SUFU* mutations, missense (c.1105G>A) and splice region (c.1157+6C>T), were identified in patients with KFS; however, it requires further research [47]. Therefore, in vertebral segmentation and limb patterning, HH signaling determines cell fates and promotes organized growth. Dysregulation of this pathway due to mutations in its components can lead to congenital deformities, including scoliosis or vertebral fusion.

### 6.4. BMP Pathway

BMP signaling plays an important role in cartilage primordia and skeletal formation, particularly limb development [46]. Cartilage production is inhibited by Noggin, the BMP antagonist, which helps to regulate proper development [62]. Additionally, the BMP pathway works synergistically with Smad and MAPK pathways in limb development [63]. Studies on *Bmp-2* and *Bmp-4* double knockout mice have shown severe bone formation defects, which highlights its critical function in vertebrae development [64].

BMP controls the proliferation of chondrocytes by the expression of SOX9, a transcription factor responsible for the regulation of cartilage matrix genes such as *COL2A1* and *COL11A1* [65]. A specific *SOX9* gene mutation (c.1405A>G) was detected in patients with congenital scoliosis [48]. Regarding the role of *COL11A2* in vertebral development, vertebral fusions were observed in mutant zebrafish models with nonsense mutation or gene deletion [66].

Other mutations of the BMP pathway connected with CSDs are missense variants of *GDF3* and *GDF6* observed in KFS [67]. GDF3 and GDF6 proteins belong to the TGF-β superfamily and play important roles in skeletal and joint formation during embryogenesis. The mutations in *GDF3* and *GDF6* genes disrupt chondrocyte differentiation and proliferation, required for the segmentation of the vertebrae [67].

### 6.5. TGF-β Pathway

Similarly to the BMP pathway, TGF-β signaling is required for skeletal formation. The TGF-β signaling pathway is involved in the development of intervertebral disks (IVD) and vertebral bodies. The TGF-β cascade regulates cell proliferation, differentiation, and synthesis of components of the intercellular substance of the IVD [68]. Disrupted TGF-β signaling due to mutations in *TGFBR2*, *SMAD2*, *SMAD3*, and other genes is associated with spinal deformities [69,70]. One research study confirmed that the deletion of *Tgfr2* in mice leads to disrupted TGF-β signaling, which results in short limbs and joint fusion [69]. TGF-β signals through a canonical pathway involving SMAD2 and SMAD3 proteins. SMAD2 plays a prominent role compared to SMAD3 in non-hypertrophic chondrocytes in the growth plate and elevated Indian hedgehog RNA levels [70]. The development and maintenance of IVDs and vertebral bodies depend heavily on the TGF-β signaling pathway. Therefore, mutations in genes interacting with this pathway can lead to skeletal abnormalities, including spinal deformities and joint fusion.

## 7. Early Diagnosis of CSDs

There are currently no direct treatments for congenital scoliosis and other CSDs, but early diagnosis through the study of the genetic basis can help in the prognosis of severity and confounding complications, such as neurological deficits, respiratory issues, cardiovascular abnormalities, and musculoskeletal complications. Knowing which genes and signaling pathways play a role in the formation of the spine in the embryo makes prenatal testing possible. Diagnosis of monogenic disorders, such as Tay–Sachs, fragile X syndrome, and cystic fibrosis, has traditionally relied on risky invasive tests, which is why non-invasive prenatal testing (NIPT) based on cell-free fetal DNA (cffDNA) present in maternal plasma is preferred [71]. Knowing which genes and signaling pathways are involved in the formation of the vertebral embryo makes it possible for early diagnosis of CSDs using NIPT.

Although a genetic cure for CS remains elusive, advances in genetic technology offer possibilities for future therapeutic interventions, such as gene editing and stem cell therapy. A successful case of CRISPR-Cas9-based treatment, with no evidence of off-target editing, in a genetic disorder, transfusion-dependent β-thalassemia (TDT) and sickle cell disease [72]. The use of CRISPR-Cas9 in congenital scoliosis is challenging due to the nature of the disease. Because congenital scoliosis and other spinal deformities are present from birth and often result from abnormal development of the vertebrae in the embryo, CRISPR-Cas9-based treatments will likely need to be administered early in pregnancy to correct the formation of the vertebrae. This in utero gene editing approach comes with ethical and technical risks. Because CRISPR permanently alters DNA, unintended consequences could have long-term developmental consequences [73]. The risk-benefit ratio will need to be carefully assessed, especially given that some forms of congenital scoliosis can be treated with surgery and supportive care.

## 8. Limitations and Future Perspectives

The genetic-based classification of CSDs can enable accurate diagnosis, which is a major step forward in understanding the causes of vertebral malformations and their treatment. The proposed classifications of CSDs based on developmental stage specificity and signaling pathways support a molecular-genetic approach to diagnosis and research. However, there are some limitations to these proposed categories of CSDs. Despite advancements, there are many CSD cases that do not have known genetic mutations. The existence of such cases requires further study and research beyond a genetic basis. One of the possible causes is epigenetic modifications, especially DNA methylation. In patients with CS, hypomethylation was detected in *COL5A1*, *GRID1*, *RGS3*, *SORCS2*, and *ROBO2*, while hypermethylation was noted in *GSE1*, *IGHG1*, *IGHM*, *IGHG3*, *KAT6B*, *TNS3*, and *RNF213* [28].

Classification of CSDs based on genetic background does not reflect environmental and epigenetic causes. Since cervical fusions seen in KFS are also observed in fetal alcohol syndrome, alcohol can impact the vertebral formation of the embryo [74]. Exposure of the embryo to valproic acid used for psychiatric and neurological disorders is associated with congenital malformations such as lumbar vertebral fusion and thoracolumbar scoliosis [75]. Another teratogenic effect is maternal diabetes, and a child born to a mother with diabetes has craniofacial anomalies, particularly fused cervical vertebrae [76].

## 9. Conclusions

In conclusion, the genetic-based classification of congenital spinal deformities (CSDs) marks a pivotal advancement in the understanding and management of these complex conditions. While current classifications provide significant insights into the developmental and molecular basis of CSDs, they remain limited by incomplete understanding of epigenetic, environmental, and multifactorial contributions. Future research should prioritize the integration of advanced genomic techniques, such as single-cell sequencing and transcriptomics, to uncover the interplay between gene expression patterns and environmental exposures during embryogenesis. Moreover, the potential role of epigenetic modifications, such as DNA methylation and histone acetylation, warrants further investigation as these factors may bridge the gap between genetic predisposition and phenotypic expression. Advances in non-invasive prenatal testing (NIPT) and in utero therapeutic interventions offer transformative opportunities for early diagnosis and targeted treatment, but ethical and technical challenges must be addressed. Collaborative, multidisciplinary efforts that incorporate bioinformatics, developmental biology, and clinical genetics will be essential for refining classifications and developing personalized therapeutic strategies. Expanding knowledge of signaling pathways and their crosstalk, as well as leveraging emerging tools like CRISPR-Cas9 for functional studies, will further enhance our ability to translate genetic discoveries into clinical applications. This comprehensive approach holds promise for improving diagnostic accuracy, therapeutic outcomes, and the overall quality of life for patients with CSDs.

## Figures and Tables

**Table 1:** Summary of established classifications for CSDs.

Winter et al. (1983) [6]	Kawakami et al. (2009) [9]	Kawakami et al. (2018) [10]
**Formation failure** Improper vertebral formation. Includes WSV, HV, BV, and VA.	**Solitary simple (SS)** Abnormal vertebrae with formation disorder. Includes HV, WSV, and BV.	**Formation failure** Improper vertebral formation. Includes WSV, HV, BV, and VA.
**Segmentation failure** Partial or complete fusion of two or more vertebrae. Includes unilateral unsegmented bars and block vertebrae.	**Multiple simple (MS)** Multiple anomalies of vertebrae, not related to anterior/posterior structure. Includes WSV, HV, BV, discrete, adjacent anomalies.	**Coupling failure** False coupling of somites resulting in contralateral HV formation.
**Mixed type** Both formation and segmentation failures or anomalies not fitting descriptions.	**Multiple complex (MC)** Formation defects with some segmentation defects.	**Segmentation failure** Partial or complete fusion of two or more vertebrae. Includes unilateral unsegmented bars and block vertebrae.
	**Segmentation failure (SFK)** Only covers segmentation failure.	**Mixed type** Covers coupling failure with segmentation or formation defects.

WSV: wedge-shaped vertebrae; HV: hemivertebrae; BV: butterfly vertebrae; VA: vertebral aplasia; SS: solitary simple; MS: multiple simple; MC: multiple complex; SFK: segmentation failure.

**Table 2:** Genes involved in CSDs, their biological function, and their effect on CSDs.

Gene	Biological Function	Effect on CSDs
*SHH*	Regulates sclerotome formation, promotes cartilage differentiation.	Mutations disrupt sclerotome development, leading to vertebral segmentation defects.
*PAX1*	Controls sclerotome differentiation into chondrocytes.	Defects cause improper vertebral segmentation and cartilage formation abnormalities.
*PAX9*	Works with the PAX1 protein in sclerotome development.	Mutations lead to the absence of vertebral column bones and IVDs.
*HOX*	Provide anterior–posterior positional identity.	Alterations disrupt vertebral patterning, leading to abnormalities in vertebral size and alignment.
*TBX6*	Regulates differentiation of paraxial mesoderm into somites.	Mutations linked to congenital scoliosis, causing vertebral segmentation defects.
*DLL3*	Involved in Notch signaling and somitogenesis.	Defects lead to SCD and improper vertebral segmentation.
*MEOX1*	Required for somite development and sclerotome polarity.	Mutations result in KFS, affecting vertebral and rib formation.
*BAPX1*	Regulates axial skeleton development and chondrogenesis.	Deficiencies contribute to vertebral fusion and skeletal abnormalities.
*JAG1*	Part of the Notch signaling pathway, regulates somitogenesis.	Mutations associated with Alagille syndrome, affecting vertebral segmentation.
*BAZ1B*	Involved in Wnt/β-catenin signaling.	Mutations associated with congenital vertebral fusion and KFS.
*KMT2D*	Transcriptional regulator linked to skeletal development.	Mutations cause multiple contiguous cervical fusion abnormalities.
*SOX9*	Transcription factor involved in chondrogenesis.	Mutations impair cartilage development, leading to CSDs.
*FZD6*	Wnt signaling receptor, involved in neural tube development.	Mutations cause neural tube defects and congenital spinal abnormalities.
*VANGL1*	Regulates planar cell polarity and neural tube closure.	Defects lead to spina bifida and other vertebral segmentation issues.
*SUFU*	Negative regulator of HH signaling.	Mutations affect chondrocyte differentiation, potentially contributing to KFS.
*COL11A2*	Essential for cartilage matrix formation.	Mutations cause vertebral fusion and skeletal defects.
*GDF3*	Member of the TGF-β superfamily, regulates skeletal formation.	Mutations disrupt chondrocyte differentiation, leading to vertebral segmentation defects.
*GDF6*	Involved in joint and vertebral development.	Deficiencies result in skeletal deformities such as KFS.
*TGFBR2*	Mediates TGF-β signaling, crucial for vertebral body formation.	Mutations lead to joint fusion and skeletal abnormalities.
*SMAD2/* *SMAD3*	Key players in the TGF-β pathway, regulating cartilage formation.	Disruptions result in vertebral segmentation defects and IVD malformations.

IVD: intervertebral disk; SCD: spondylocostal dysostosis; KFS: Klippel–Feil syndrome.

**Table 3:** Signaling pathways with associated genes that are linked to the development of CSDs.

Signaling Pathway	Key Functions	Key Genes	Associated CSDs
Wnt	Regulates cell proliferation (canonical) and cell polarity (non-canonical).	*BAZ1B*, *KMT2D*, *SOX9*, *FZD6*, *VANGL1*	Congenital vertebral fusion, KFS, neural tube defects, spina bifida
Notch	Controls intercellular interactions and somitogenesis; regulates vertebral segmentation.	*RIPPLY2*, *TBX6*, *JAG1*	KFS, CS, Alagille syndrome
Hedgehog	Determines cell fate in vertebral segmentation and limb patterning; maintains and repairs skeletal tissue.	*PAX1*, *SUFU*	Scoliosis, vertebral fusion, neural tube defects
BMP	Regulates limb development and chondrocyte proliferation.	*SOX9*, *COL2A1 SOL11A1*, *GDF3*, *GDF6*	CS, vertebral fusions, skeletal abnormalities
TGF-β	Required for skeletal formation, vertebral body, and IVD development.	*TGFBR2*, *SMAD2*, *SMAD3*	Spinal deformities, joint fusion, disrupted IVD development

KFS: Klippel–Feil syndrome; CS: congenital scoliosis; IVD: intervertebral disk.
